# Hydrochemical characteristics and genesis model of Yaoshuitan geothermal field in Huangzhong District, Xining City

**DOI:** 10.1038/s41598-025-07590-6

**Published:** 2025-07-02

**Authors:** Qin Guangxiong, Xie Xiandong, Zhao Zhen, Yan Baizhong, Xiao Shanhu

**Affiliations:** 1 Qinghai 906 Engineering Survey and Design Institute Co. Ltd, Xining, 810000 China; 2 Qinghai Engineering Research Center of Geoenvironment Protection and Geohazard Prevention, Xining, 810000 China; 3 Key Laboratory of Environmental Geology of Qinghai Province, Xining, 810000 China; 4 Hebei GEO University, Shijiazhuang, 050031 China

**Keywords:** Hydrochemical characteristics, Isotopes, Drilling, Geophysical exploration, Genesis model, Yaoshuitan geothermal field, Geothermal energy, Hydrology

## Abstract

The Yaoshuitan geothermal field in Huangzhong District, Xining City, Qinghai Province, is located at the southern edge of the Xining Basin. The geothermal resources in this area belong to the uplifted mountain and limestone type, which are entirely different from the geothermal resource’s characteristics in the northern part of the Xining Basin. Studying its formation mechanism is of significant importance for guiding the search for geothermal resources in limestone regions. The results indicated that: (1) The study area is characterized as a small-scale, low-to-medium temperature geothermal field. Groundwater from wells YST1 and YST2 was measured at 41 °C, and the hydrochemical composition was classified as the HCO_3_-Ca·Mg type. (2) The geothermal water is primarily replenished by atmospheric precipitation from the southern Laji Mountains at elevations ranging from 3619.52 to 3797.52 m. The temperature of the thermal reservoir ranges from 56.52 to 67.34 °C. (3) The genesis model is as follows: The heat source is derived from heat flow in the lower crust and upper mantle. Due to the unique geological conditions of the region, no effective cap rock is present in the Yaoshuitan geothermal field. The thermal reservoir is primarily composed of carbonate rocks of the Kesuer Formation within the Jixian System.

## Introduction

Geothermal resources are renewable, low-carbon, environmentally friendly, stable, and capable of diverse applications. They are among the most practical and competitive resources in renewable energy^1,2^.The numerous mountainous regions of Qinghai Province, with their unique geological structure, have created favorable conditions for the formation and storage of geothermal water through the accumulation of Mesozoic-Cenozoic deposits. This also provides a substantial geothermal reservoir capacity^3,4^.The Xining Basin is located between the Loess Plateau and the Qinghai-Tibet Plateau, two distinct tectonic and geomorphological units. It serves as a transitional and connecting zone between these two landforms, and is classified as a Block edge uplift and central depression basin. The geothermal area of the basin spans 1143.0 km^2^, with a geothermal natural resource volume of 105.48 × 10^6^m^3^/a and a basic thermal energy of 238.69 kJ^5^. Geothermal water in the Xining Basin typically exhibits high mineralization, with a water chemistry type of SO_4_·Cl–Na. The thermal reservoirs are primarily composed of Cenozoic clastic rock-type pore and fracture reservoirs^6,7^. The Yaoshuitan area in Huangzhong District, Xining City, is located in the geothermal zone of the Laji Mountain fault zone, along the southern margin of the Xining Basin. Secondary NE-trending faults are distributed along the main NWW-trending controlling fault in the area. The geothermal anomalies are primarily concentrated at the intersections of these faults. The geothermal field of Yaoshuitan has a water chemistry type of HCO_3_-Ca·Mg, with a mineralization degree of less than 3 g/L. The heat reservoir consists of limestone, which is a carbonate rock fissure-cavern type heat reservoir^8^. The geothermal field at Yaoshuitan differs from those within the Xining Basin in both its water chemistry characteristics and geothermal genesis, especially showing significant differences compared to the high mineralization geothermal waters in the Xining Basin. This indicates that the genesis model of the Yaoshuitan geothermal field is somewhat typical, and it is of great significance for understanding the formation of geothermal fields influenced by deep major fault structures and the convective-type geothermal fields associated with uplifted mountains in the study area.

By studying the water chemistry characteristics of geothermal water, the genesis of the geothermal water can be effectively revealed. Lu et al. studied the geothermal fluids in the northern region of Zhangqiu using hydrogeochemical and isotope analysis methods^9^. Du et al. studied the geothermal waters in the Qingshankou Formation in the southern Songliao Basin, noting that the primary ionic components of the geothermal water in the area are Na^+^, Cl^−^, and HCO_3_^−^, with recharge sources coming from atmospheric precipitation and primary sedimentary water^10^. Deng et al. through water chemistry analysis of the Guizhou Mercury Cave geothermal field, combined with hydrogeochemical modeling, determined the recharge sources, water–rock reactions, and genesis of the geothermal water^11^. Avsar and Altuntas, based on the water chemistry and isotope composition of the Umut geothermal field, identified the genesis of the geothermal water, concluding that the mixing of shallow groundwater is the main factor influencing the characteristics of the geothermal water^12^. Landa-Arreguin et al. studied the water chemistry of the high-temperature geothermal field in Mexico, analyzing that the geothermal water chemistry genesis is primarily controlled by cation exchange, silicate, and carbonate rock alteration, while also being influenced by the mixing of shallow Na–HCO_2_ type waters^13^

In addition to studying the geothermal water genesis through water chemistry, many scholars have constructed geothermal genesis models by examining factors such as the heat source, geothermal reservoir, reservoir cap, and heat transfer pathways to explore the origin of geothermal energy^14^. Yan et al. established a conceptual model for the genesis of geothermal water in the basin-type geothermal system of the Changbai Mountain basalt area^15^. Mo et al. used methods such as gravity measurements and Controlled Source Audio Magnetotellurics (CSAMT) to investigate the geothermal reservoir formation conditions in the northern Liuzhou region of Guangxi, and developed a geothermal genesis model^16^. Wang et al. by establishing a geothermal genesis conceptual model, determined that the Yangmeichong geothermal system is of the fault convection type. The geothermal water moves downward through fault zones and rock pores as water-conducting pathways, driven jointly by hydraulic and thermal forces^17^. Song et al., in their study of the southwestern Tibet Plateau’s fault structures and volcanic activity characteristics, summarized the sources, reservoirs, cap rocks, and pathways in the region, establishing a geothermal genesis conceptual model^18^. Zhu et al. developed a conceptual model for geothermal genesis in the northern Songliao Basin, pointing out that the primary heat source is the large geothermal flux from the upper mantle, which is also influenced by fault structures^19^.

The Yaoshuitan geothermal field in Huangzhong District, Xining City, is located at the southern edge of the Xining Basin. It lies in the hilly area of the middle and upper reaches of the Nan Chuan River Valley, at the northern slope of the Laji Mountain. The controlling fault at the southern edge of the Xining Basin is the North Laji Mountain Fracture. Li et al. conducted a preliminary investigation into the hydrochemical characteristics and the origin of the geothermal water in the Yaoshuitan geothermal field^20^. However, there is a lack of systematic research on the control of fault structures on the geothermal water in the region and the geothermal genesis model. The paper aims to use a combination of hydrogeochemistry, geophysical exploration, and geothermal drilling methods to thoroughly investigate the hydrochemical characteristics of the Yaoshuitan geothermal field. The goal is to determine its chemical composition, recharge sources, recharge elevation, geothermal reservoir temperature, and circulation depth. Based on geophysical exploration and geothermal drilling, the study will identify the heat source, cap rock, geothermal reservoir, and the role of fault structures in controlling the geothermal system. The research will help establish the genesis model for the geothermal field and contribute to the understanding of geothermal resource genesis in the Xining Basin, particularly in relation to the geothermal genesis influenced by deep major fault structures.

## Study area

**Fig. 1:**
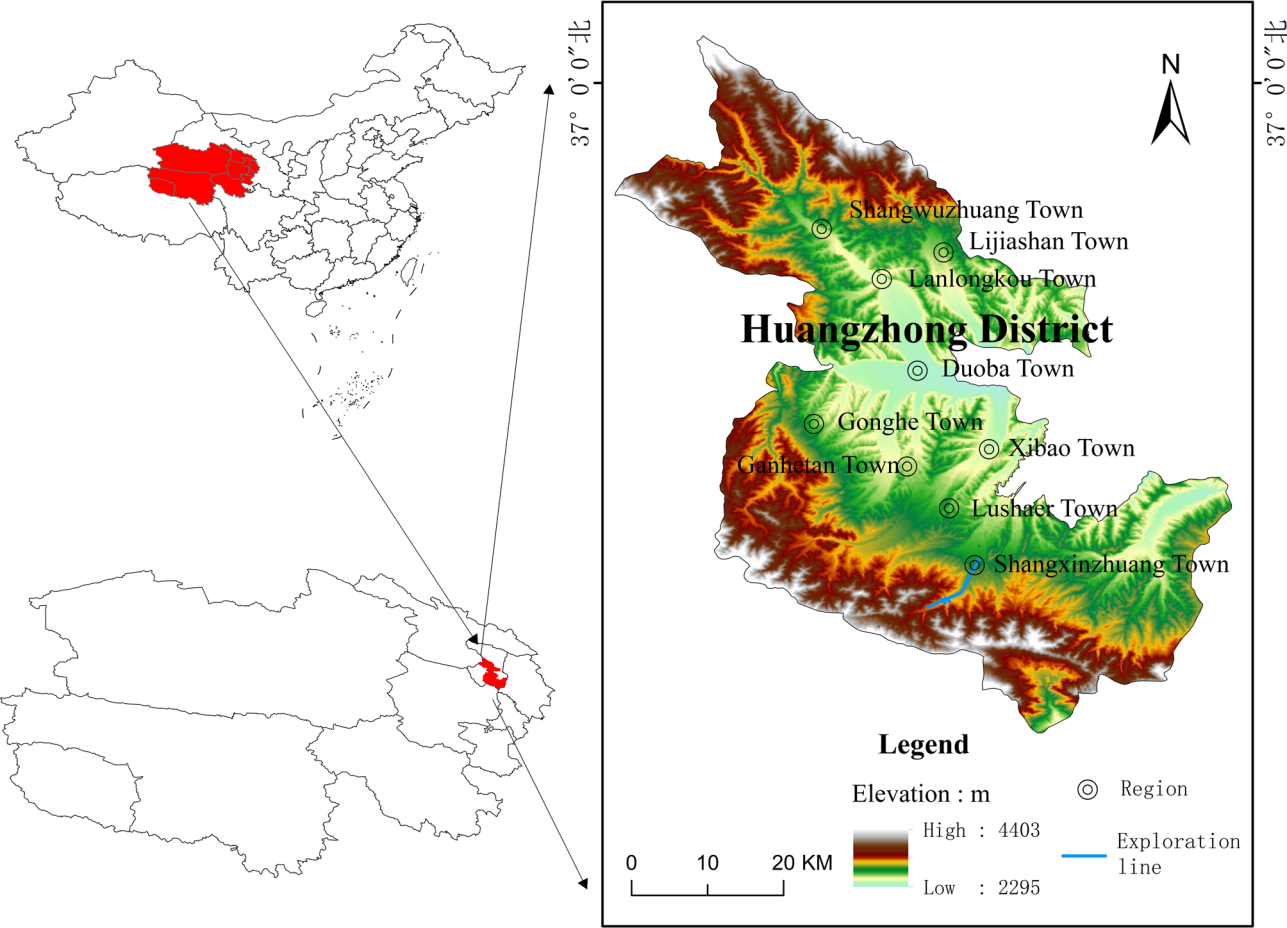
Topographic and geologic map of the study area. The map was created with ArcGIS 10.8.1 (https://www.esri.com/en-us/arcgis/products/arcgis-desktop/resources) and is based on the standard map No. GS(2024)0650 provided by MapWorld of the National Platform for Common GeoSpatial Information Services ( https://map.tianditu.gov.cn/). No modifications were made to the base map.

The study area located in Shangxinzhuang, Huangzhong District, at the southern edge of the Xining Basin, approximately 25 km from the urban center of Xining. The region has an arid to semi-arid continental climate, with an average annual temperature ranging from 7 to 9 °C, annual precipitation of 537.6 mm, and evaporation reaching 1700 mm. Precipitation is concentrated from July to September, accounting for 55% of the total annual rainfall. The area lies at the southern edge of the Xining Basin, in the hilly zone of the northern slope of the Laji Mountain and the middle and upper reaches of the Nan Chuan River Valley. The terrain in the region is complex and diverse. From south to north, the landscape consists of medium to high mountains made up of metamorphic rocks, low mountains and hills covered with loess, and river valley plains. The elevation of the region is approximately 4000 m (Fig. 1).

The primary stratigraphic units in the study area include the Quaternary, Neogene, Paleogene, Cambrian, Jixian and Changcheng systems. The Paleogene is distributed in the northern part of the study area, where the predominant lithologies are mudstone and glutenite, characterized by a sandy texture with both argillaceous and calcareous cementation. In the central region of the study area, the Jixian system of the Middle Proterozoic is exposed, featuring crystalline limestone, dolomite, and marble of the Kesuer Formation. To the south, the Changcheng system is represented by the Qingshipo Formation, followed by the Cambrian Maojiagou Formation. The lithology of the Qingshipo Formation mainly consists of gray, thin-bedded, silty slate interbedded with metamorphosed sandstones, calcareous slates, phyllites, and crystalline limestones. The Maojiagou Formation is dominated by intermediate base rocks, with gray-green to dark green neutral lava exhibiting granular metamorphic textures and dense, massive lava structures. The Quaternary is widely distributed in the Nan Chuan River Valley and the hilltops on either side of Yaoshuitan. The Upper Pleistocene is predominantly composed of mudstone-siltstone layers and yellow sandy clay, while the Holocene consists mainly of modern riverbeds, alluvial fans, terrace-alluvial deposits, and aeolian loess (Fig. 1).

The geothermal anomaly zone of Yaoshuitan hot water aquifer lies within a thick carbonate rock formation of the arch uplift area. Four NE-trending extensional tensile-torsion fractures are developed in this region: Fracture F4-1, Fracture F4-2, Fracture F4-3, and Fracture F4-4, as well as two NNW-trending Compression-torsion fractures: the Northern Laji Mountain Fault Zone and Fracture F3. The Northern Laji Mountain Fault Zone consists of several discontinuous fractures, starting from the Shangen Village area near the Riyue Mountain Pass in the west and extending eastward along the southern margin of the Xining Basin. This fracture exhibits clear segmentation. At the northern entrance of Laji Mountain, near the Beimen Gou, the fracture branches into two, with one branch terminating at Shiju Village and the other at Qingshipo. A southward convex arc-shaped section of the fracture near Shangxinzhuang terminates eastward at Shibiyan. The fracture shifts northward and an NWW-trending section stops at Shangzhangfang, south of Shagou Village. Further north, near Yingpo Village, a slight convex arc is developed, and the fracture terminates at the Hongyazi area after passing through Zhongba and Tangeryuan. The fault zone extends approximately 230 km, with the strike changing from NW60° in the west to nearly EW and NNW directions in the east. To the south lies the towering Laji Mountain Range, while to the north is the Xining Basin, marking an important boundary fracture between the basin and the mountain, with a significant difference in topography on both sides. Fault F3, a secondary fault of the La Bei Fault Zone, is a nearly N-S trending compressional strike-slip fault, located on the upper plate of the La Bei Fault along the Xiao Maji River. The Northern Laji Mountain Fault Zone, composed of several discontinuous faults, was most active during the Paleogene and early Quaternary periods. Activity decreased during the late Quaternary, but some segments remained active into the late Pleistocene and even into the Holocene.

**Fig. 2:**
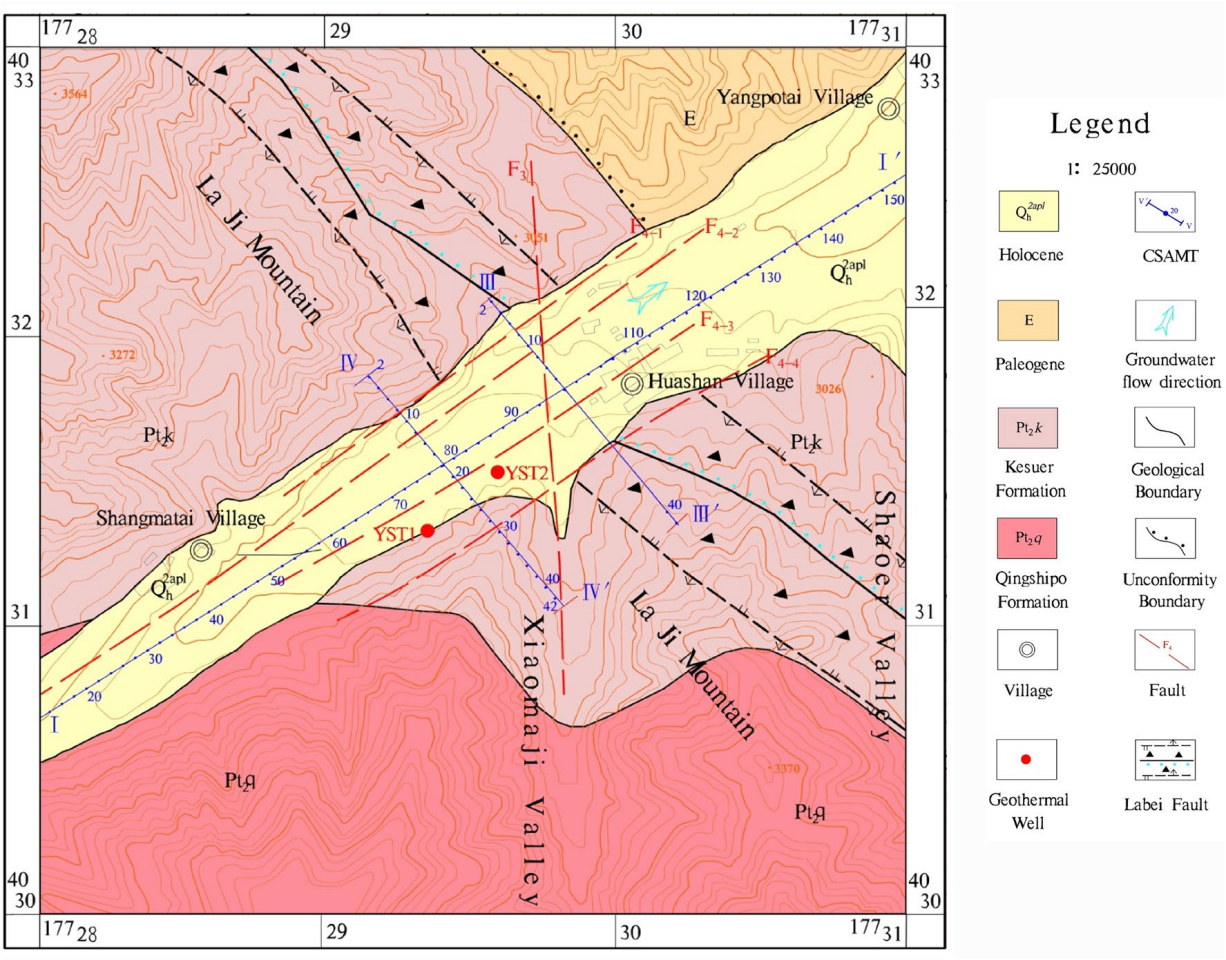
Crustal and deep tectonic zoning of the Xining Basin(contours/km).

The crustal structure and geophysical anomalies in the Yaoshuitan area of Huangzhong District are highly prominent. Gravity and magnetic anomalies exhibit a nearly north–south distribution within the basin. In terms of crustal structure, the Yaoshuitan area lies at the junction of the Xining-Tongren mantle slope zone, extending in an NNW direction, and the Haiyan-Guinan mantle platform zone. From west to east, the upper mantle depth increases from 55 to 53.5 km, with a variation of approximately 1.5 km. The upper mantle’s surface shows a steep slope from west to east, with the Moho discontinuity continuously rising, indicating the presence of a low-resistance layer that forms an upward arch within the crust. Due to its location in the uplift zone of the upper mantle, the crust in the Yaoshuitan area is elevated by up to 1.5 km relative to the Haiyan-Guinan mantle platform zone, where the depth is 55 km. The geothermal anomaly area in the Yaoshuitan region of Huangzhong district generally extends along the northeast-southwest-oriented La Bei Fault, forming a band-shaped distribution. It is approximately 800  m in length from east to west and 600 m in width from north to south. The primary geothermal reservoir consists of crushed breccia from the La Bei Fault (Fig. 2).

**Fig. 3:**
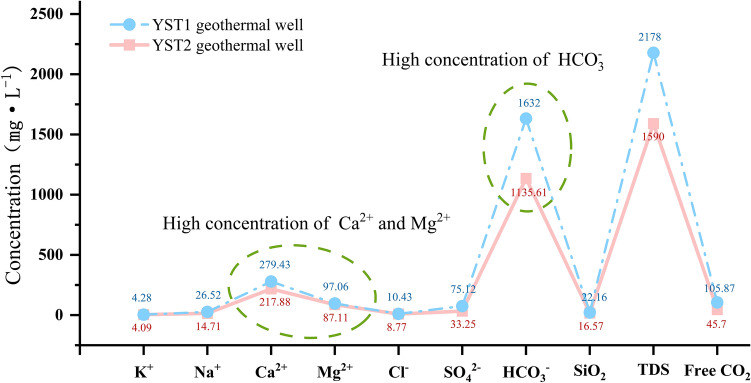
Isothermal contour map of ground temperatures in the study area.

The shallow geothermal field of the Yaoshuitan geothermal area exhibits a pronounced structural control on heat distribution. Ground temperatures at depths of 50 m (ranging from 20 to 35 °C) and 150 m (ranging from 30 to 40 °C) are both distributed in NE–SW-oriented belt-like patterns, aligned with the northern margin fault of the Laji Mountain and associated NE-trending secondary faults. Borehole YST1 is located within the high-temperature core zone at a depth of 150 m, while borehole YST2 lies within the high-temperature core at 50 m depth. The overall configuration of isotherms reveals a south-high to north-low gradient (Fig. 3).

## Research methods

To investigate the geothermal geological conditions in the study area and the impact of the main controlling fracture (the northern boundary fracture of the Laji Mountain) on geothermal wells, geophysical exploration and geothermal drilling methods were employed. Three Controlled Source Audio Magnetotellurics (CSAMT) profiles were established in the area: Profile I-I′, Profile II-II′, and Profile III-III′. The I-I′ profile is 2.5 km long, with 50 measurement points; the II-II′ profile is 1.05 km long, with 21 measurement points; and the III-III′ profile is also 1.05 km long, with 21 measurement points. Data processing involved filtering methods to eliminate electromagnetic interference, correcting transition zones, and performing static displacement correction. Interpretation was carried out using inversion software, which performed one-dimensional and two-dimensional continuous smoothing inversion calculations of the resistivity profile (ρ_s_) along the entire profile. The frequency domain data were converted into depth resistivity sections, and the inversion profiles were used to interpret the burial conditions and spatial distribution of the La Bei reverse thrust and the F4 extensional fault zone. The CSAMT measurements were conducted using the GDP-32II multi-functional electrical workstation, manufactured by Zonge Corporation, USA.

In addition, two geothermal wells (YST1 and YST2) were drilled in the area. The depth of well YST1 is 1403.46 m, with a hydraulic head of + 12.1 m. The flow rate at the wellhead is 1465.34 m^3^/d, and the stabilized flow rate at a depth of 58.24 m is 2378.07 m3/d, with a water temperature of 41 °C. The depth of well YST2 is 52.68 m, where geothermal water flows to the surface from the fault zone between 20.44 m and 52.68 m. The hydraulic head is + 17.5 m, with a flow rate of 14.23 L/s and a water temperature of 41 °C. Sampling tests were conducted on both YST1 and YST2, including analyses of major ion components and isotopic components. The major ion components include pH, K^+^, Na^+^, Ca^2+^, Mg^2+^, Cl^−^, SO_4_^2−^, HCO_3_^−^, SiO_2_, total dissolved solids, and free CO_2_. The isotopic components include δD, δ1^8^O, and 14 C. Additionally, geophysical logging was performed on YST1 using a PSJ-2 digital logging instrument, manufactured by Beijing Zhongdi Yingjie.

To determine the genetic model of geothermal resources in the study area, this research establishes a framework for multi-method collaborative analysis. Firstly, comprehensive analysis is conducted on data including geological conditions, geophysical exploration results, and geothermal well drilling locations within the area to identify fundamental factors governing geothermal formation, such as heat sources, cap rocks, geothermal reservoirs, geothermal gradients, and groundwater runoff pathways of the geothermal system, thereby constructing the geological framework of the geothermal model. Secondly, through analysis of chemical components and isotopes in geothermal water, the recharge sources of groundwater, circulation depth, and reservoir temperature are determined. Finally, the hydrogeochemical formation processes are revealed via multi-method collaborative analysis including inverse hydrogeochemical modeling. By integrating key factors influencing geothermal formation—such as heat sources, cap rocks, geothermal reservoirs, recharge sources, circulation pathways, and water–rock interactions—the genetic model of the geothermal system in the area is established.

## Results and discussion

### Analysis of geothermal water characteristics

#### Chemical composition of geothermal water

**Fig. 4:**
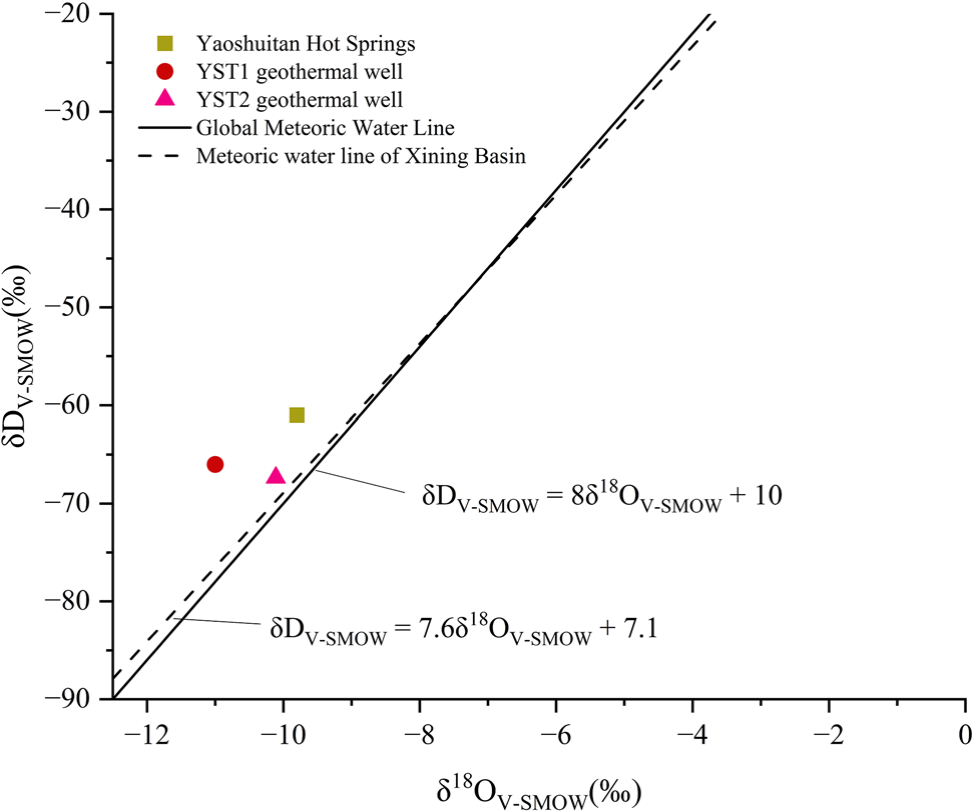
Schoeller diagram of the main water chemical composition concentrations for geothermal wells YST1 and YST2.

The analysis of the sampling results from geothermal wells YST1 and YST2 revealed that the water temperature of both wells is 41 °C, classifying them as low-temperature geothermal fields. The pH values range from 6.94 to 7.82. In terms of chemical composition, the cations are predominantly Ca2^+^ and Mg2^+^, with average concentrations of 248.655 mg/L and 92.085 mg/L, respectively. The anion is mainly HCO_3_^−^, with an average concentration of 1383.805 mg/L. The water chemistry type is HCO_3_^−^Ca·Mg, and the mineralization is between 1.590 and 2.178 g/L, classifying the water as mildly saline (Fig. 4).

#### Sources of geothermal water recharge

**Fig. 5:**
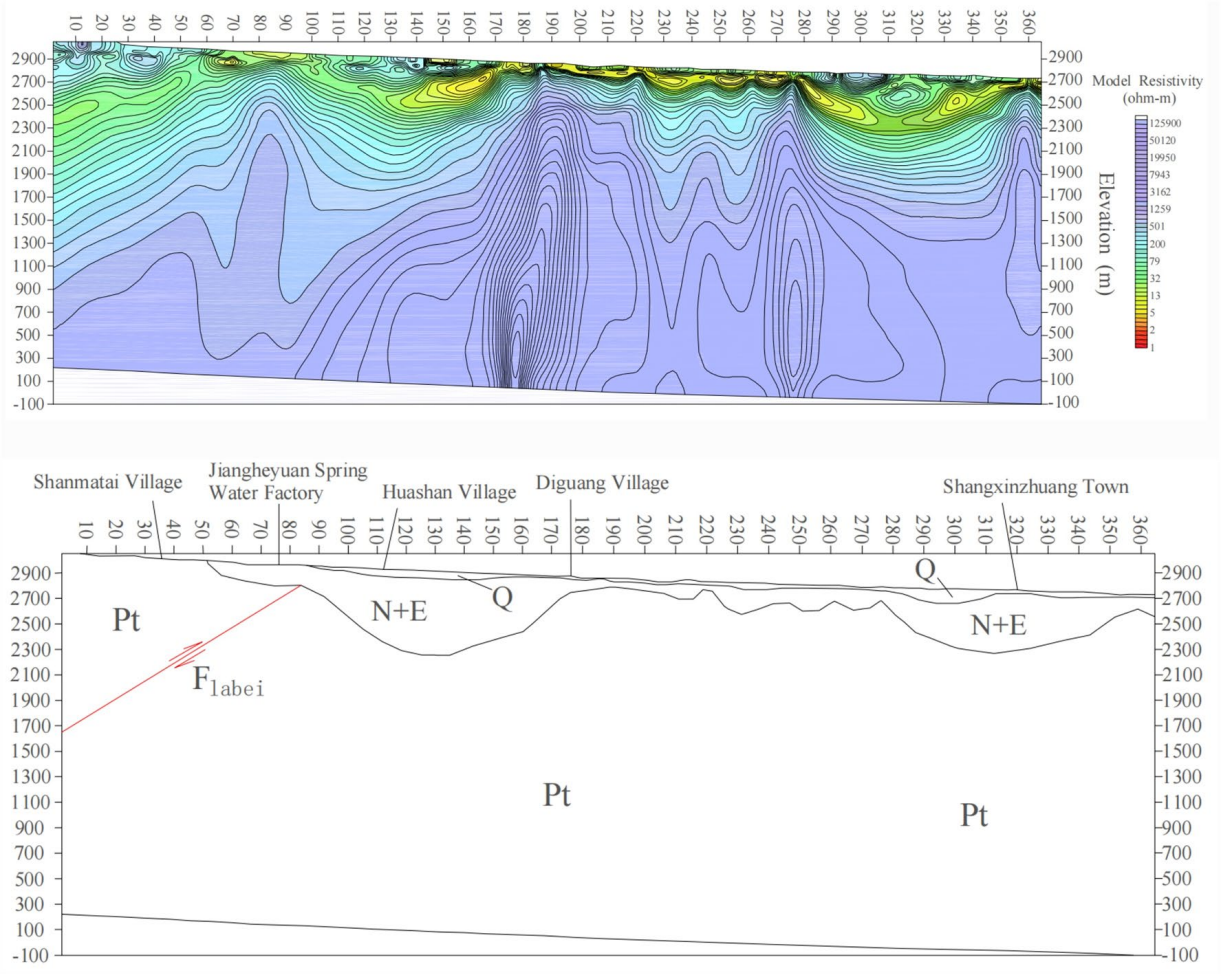
δD-δ^18^O relationship diagram of geothermal water from Yaoshuitan.

By plotting the δD-δ^18^O relationship of water samples from geothermal wells YST1 and YST2 (Fig. 5), it is observed that the global meteoric water line is δD = 8δ^18^O + 10^21^ and the meteoric water line equation for the Xining Basin is δD = 7.6δ^18^O + 7.1^22^. The hydrogen and oxygen isotope data for both geothermal wells fall near the meteoric water line, indicating that the recharge source of the geothermal water in the study area is atmospheric precipitation. The depth of well YST1 is 1403.46 m, and the depth of well YST2 is 52.68 m. The geothermal water is primarily recharged laterally from the southern mountainous area, with deeper geothermal water being influenced by secondary faults along the valleys. This leads to a younger age and a lower temperature in the deeper geothermal water compared to the upper layers (Table 1).

**Table 1:** Isotope testing results of geothermal water samples from Yaoshuitan.

Sampling location	Water type	Temperature (°C)	δ^18^O (‰)	δD (‰)
YST1 geothermal well	Geothermal water	41.0	− 11.00	− 66.00
YST2 geothermal well	Geothermal water	41.0	− 10.11	− 67.36
Yaoshuitan hot spring	Hot spring water	21.5	− 9.80	− 61.00

#### Geothermal water recharge elevation

By utilizing the elevation effect of hydrogen and oxygen isotopes, the recharge area and recharge elevation of the deep underground geothermal water in the study area are calculated using the following Eq. (1):

1 $$H = \frac{\delta G - \delta P}{K} + h$$

In the equation, H is the elevation of the recharge area, in m; h is the elevation of the sampling point, taken as 2977.518 m; δG is the δ18O value in the geothermal water, in ‰; δP is the δ18O value in precipitation (with an average δ18O value of − 6.9‰); K is the isotope altitude gradient (with δ18O of − 0.5‰/100m).

Based on the water isotope data from the YST1 and YST2 geothermal wells, the results indicate that the Yaoshuitan geothermal field is primarily recharged by atmospheric precipitation infiltration from the mountainous areas to the south, at elevations ranging from 3619.52 to 3797.52 m.

#### Reservoir temperature and circulation depth

##### Reservoir temperature

Silica in geothermal water is formed by the dissolution of quartz by hot water. This portion of the hot water does not boil upon reaching the sampling point (spring or wellhead) and can be calculated using the following equation^23^:

2 $${\text{T = }}\frac{{{1309}}}{{{5}{\text{.19 - lg(SiO}}_{{2}} {)}}} - 273.15$$

In the equation, the SiO_2_ concentration in the YST1 geothermal well is 22.16 mg/L, and in the YST2 geothermal well, it is 16.57 mg/L. Using the silica geothermometer, the reservoir temperature at 1403.46 m in the YST1 geothermal well is calculated to be 67.34 °C, which is 26.34 °C higher than the wellhead temperature. For the YST2 geothermal well at 52.68 m, the reservoir temperature is calculated to be 56.52 °C, 15.52 °C higher than the wellhead temperature.

## Circulation depth

The Yaoshuitan geothermal field is situated at the edge of the Xining Basin, where the underground hot water primarily constitutes a convective geothermal system. The circulation depth can be calculated using the reservoir temperature, average air temperature, depth of the thermocline, and geothermal gradient, as indicated in the following equation:

3 $$H = \frac{{T - T_{0} }}{{\text{g}}} + h$$

In the equation, H denotes the circulation depth (m); T is the reservoir temperature of the study area (°C); T0 is the average air temperature of the study area (°C); h is the depth of the thermocline (m), assumed to be 30 m; and g is the geothermal gradient (°C/100 m). The calculated circulation depth for the YST1 geothermal well is 3756.69 m.

### Geophysical exploration results

Based on the results of three controlled-source audio magnetotellurics (CSAMT) sounding profiles, each profile generally exhibits a three-layer H-type resistivity structure. The corresponding geological structure is characterized by a thin cap layer with alternating high and low resistivity, a low-resistivity thermal reservoir layer, and a thick, high-resistivity basement. Using known measurement points, the inversion interpretation of electromagnetic sounding yields the following resistivity values for each layer: Quaternary sediments show significant resistivity variation (10–1000 Ω m); fault fracture zones exhibit resistivities of 10–300 Ω m; and intact limestone has resistivities exceeding 800 Ω m.

**Fig. 6:**
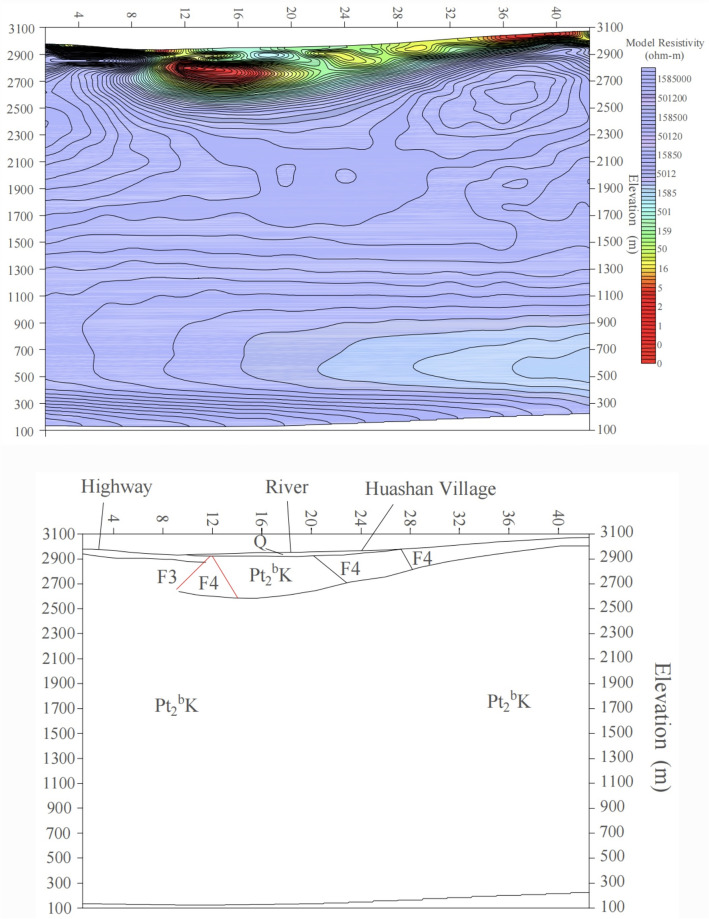
Integrated geophysical profile inversion of profile I-I′

The I-I′ profile (spanning points 2–364) is divided into two segments: points 2–168 with an azimuth of 40°, and points 170–364 with an azimuth of 23°. The profile is oriented approximately perpendicular to the fault structure along the northern margin of Laji Mountain. Inversion results indicate that the basement along I-I′ is generally shallow, with the overlying cover layer undulating moderately, reaching a maximum depth of 600 m (Fig. 6). The uppermost layer consists of Quaternary deposits, primarily composed of clayey sand, pelitic silty-sand, sandy gravels, and gravelly pebbles, with resistivities ranging from 15 to 600 Ω m and variable thickness along the profile. South of the profile (points 2–98), bedrock outcrops consist of Proterozoic formations, including crystalline limestone and dolomitic limestone. North of Point 98, the basement surface rises gradually from south to north. Based on lithological changes and low-resistivity characteristics on both sides, this reflects the composite features of the fracture zone controlling the Yaoshuitan hot springs and the northern Laji Mountain fault. The northern Laji Mountain fault is a deep-seated regional fault serving as a heat-source pathway, while the NNE-striking Shunhe Valley fault— a tensile structure—acts as a fluid conduit. Numerous hot springs form near the intersection of these two faults. According to the inversion map, the fault is a south-dipping reverse fault that acts as a groundwater barrier: the southern low-resistivity zone extends deeply along the fault plane, whereas the northern high resistivity reflects impermeable intact bedrock.

Shallow low-resistivity layers (20–200 Ω m) occur at points 98–186 and 280–356, identified as Paleogene strata extending to ~ 600 m depth. These layers induce resistivity depression in underlying strata, forming small depressions in the resistivity profile. Below lies a high-resistivity layer (> 800 Ω m, 2500–3000 m thickness), corresponding to the Proterozoic basement. Analysis of Kanya resistivity and 2D inversion results reveals distinctive stratigraphic features, with a notable south-to-north trend of decreasing resistivity, particularly near the La Bei fault. Northeast of the La Bei fault, Paleogene strata unconformably overlie the basement, with variable stratal thickness.

**Fig. 7:**
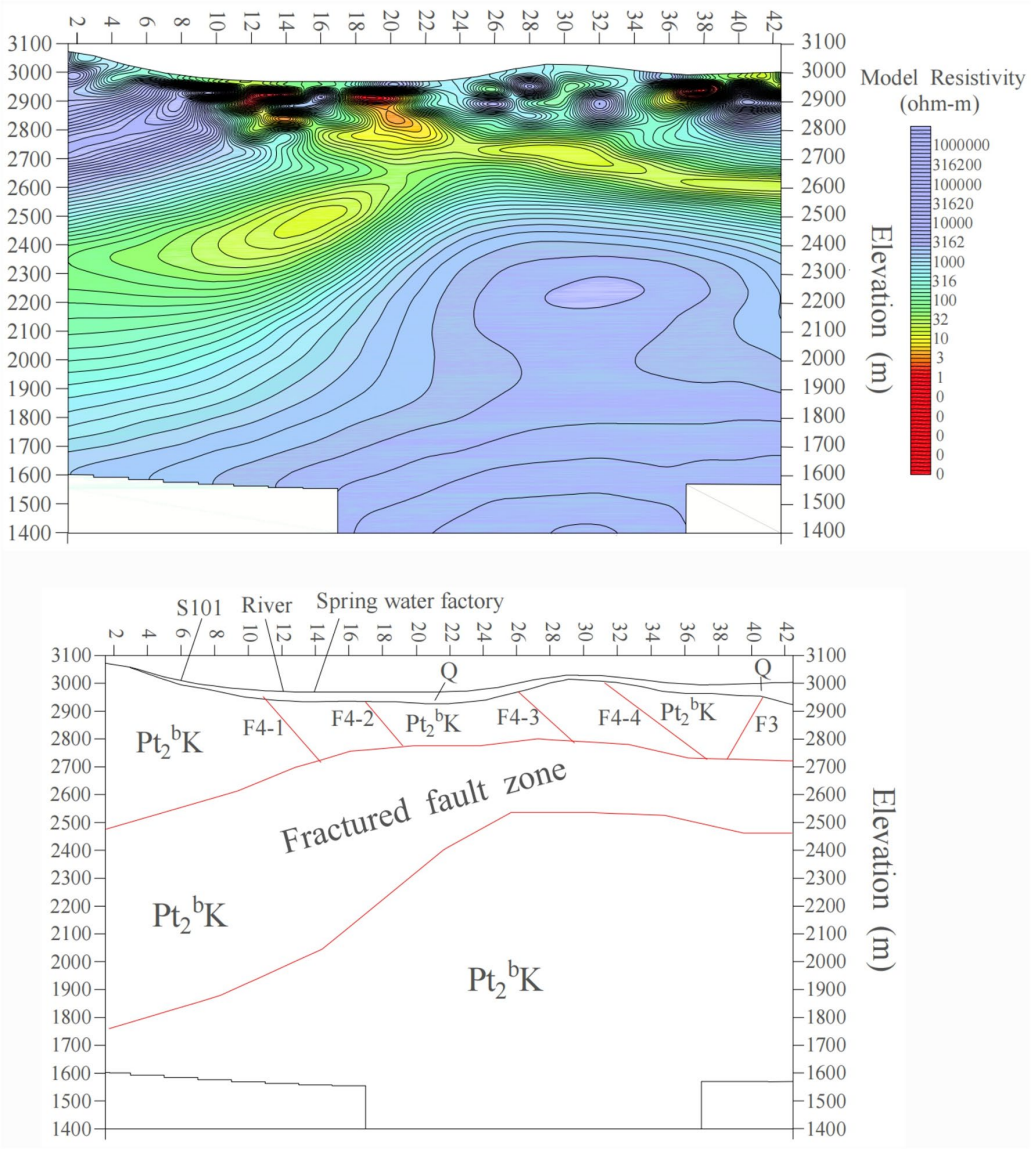
Inversion of the integrated geophysical profiles for II-II′ and III-III′ (**a**) Profile II-II′, (**b**) Profile III-III′).

The II-II′ and III-III′ profiles are located at points 78 and 98 of the I-I′ profile, respectively, and are oriented northwest, transversely crossing the valley. The II-II′ profile is perpendicular to the Dama Jigou fault, with an azimuth of 140°. Although the profile aligns with the northern Laji Mountain fault, a low-resistivity layer is evident along II-II′ on the 2D inversion diagram (Fig. 7a). On the resistivity inversion diagram between points 10–26 on the southern segment, abrupt resistivity gradients indicate the extension of the F4 fault. However, this fault zone occurs at relatively shallow depths, with the resistivity interface at point 31 interpreted as the F3 fault intersection. Both fault systems appear shallow on the 2D plot, showing a trend of gradual shallowing, with the deepest penetration at ~ 100 m. A horizontal low-resistivity layer (100–300 m depth) likely represents the La Bei fault fracture zone. Below 300 m, a high-resistivity layer exhibits a horizontal stratified structure with resistivities up to several thousand Ω m, corresponding to intact bedrock. Between points 2–10, a thin low-resistivity layer (maximum thickness ~ 10 m) corresponds to Quaternary loose sediments. South of point 10, the low-resistivity zone deepens abruptly, coinciding with the extensional F4 fault. This resistivity anomaly, caused by faulting and thermal water infiltration, influences depths up to ~ 300 m. Southward, the low-resistivity layer shallows as the basement uplifts. Inversion results indicate that fault-induced low-resistivity zones are shallow here, suggesting basement uplift north of the Laji Mountain northern boundary fault and diminished fault influence. Thermal water is predominantly hosted in strata south of the fault (Fig. 7a).

The III-III′ profile (azimuth 140°) exhibits a generally horizontal trend, with the basement deepening northward and shallowing southward. The curve type transitions from KH-type in the north, to H-type in the center, and finally to A-type in the south, reflecting lithological and stratigraphic variations. The first resistivity layer (15–600 Ω·m) comprises Quaternary clayey sand, gravels, and pebbles, with thickness ranging from 0 to 30 m. The second layer is a low-resistivity zone (a few Ω m to 300 Ω m), primarily associated with faulted and water-saturated Proterozoic fracture zones, interpreted as the La Bei fault zone controlling the Yaoshuitan hot springs. Below this lies a high-resistivity layer (> 800 Ω m, 2500–3000 m thickness), corresponding to Proterozoic metamorphic basement (Fig. 7b).

Through geophysical exploration and analysis, it has been concluded that there are three main sets of fractures in the study area that control the formation of geothermal water. Deep and large fractures conduct heat and heat the groundwater, conductive fractures serve as channels for groundwater flow and accumulation, and impermeable fractures create geothermal water enrichment. Among them, the Laji Mountain northern boundary fault is the controlling fault on the southern margin of the Xining Basin, with differential uplift and subsidence movements of fault blocks dominating during the neotectonic period. The trace of the Laji Mountain northern boundary fault is particularly evident near the Gui De Gorge, south of Shangxinzhuang. The fault can be divided into two segments with different motion characteristics, with Shangxinzhuang as the boundary. The eastern segment (to the east of Shangxinzhuang) is primarily characterized by vertical displacement, with poor linear features and a gently undulating shape, reflecting strong compressive reverse thrust characteristics. The western segment (to the west of Shangxinzhuang) shows clear linear features and is mainly characterized by reverse strike-slip movement, often forming synchronous bends in the drainage system and faulted ridges, with a maximum displacement of 1100 m. The F3 fault, along with the La Bei fault zone, has created mylonite in the Da Ma Ji River valley due to the strike-slip and compressional-torsional geological processes. This is an important factor in the large-scale exposure of the Yaoshuitan hot spring and forms an insulating and impermeable structure, providing favorable conditions for hydrothermal activity and surface discharge. It is one of the decisive factors in the formation of the Yaoshuitan geothermal field. It is evident that geothermal or thermal anomalies are distributed near the four NE-trending extensional-strike-slip fault zones in the area, indicating that these faults cut deep and provide favorable channels for the migration of thermal fluids.

### Geothermal drilling results

**Fig. 8:**
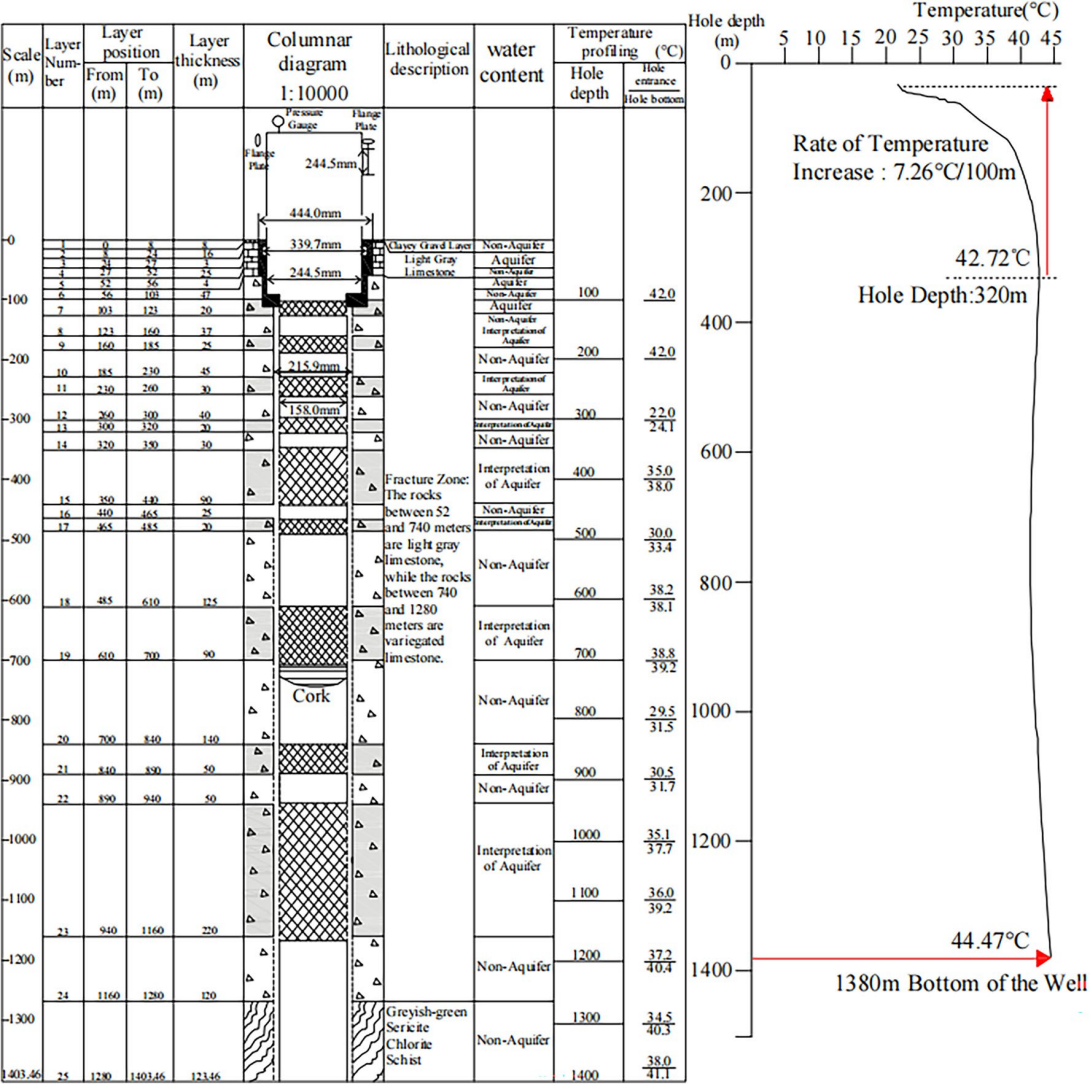
Borehole lithology and temperature profile of geothermal well YST1 in the Huangzhong district Yaoshuitan geothermal field.

Through the analysis of rock cuttings logging, geophysical logging, and regional stratigraphic correlation during the drilling process, the borehole profile of the YST1 geothermal well was established (Fig. 8). The exposed strata in the YST1 geothermal well include Quaternary and Proterozoic formations. The Quaternary upper Pleistocene (Q_4_^apl^) consists of a thin layer of yellow-gray loess at the top and a black silty gravel layer at the bottom. The thickness exposed in the borehole is 8.0 m, which forms the foothill alluvial fan along both sides of the Ma Ji River. The Proterozoic formation includes the Kesuer Group limestone (Pt_2_bK) and Kesuer Group schist. The upper Proterozoic Kesuer Group limestone is light gray, with a massive structure, and shows strong effervescence when reacted with hydrochloric acid. The thickness exposed in the borehole is 95.0 m, with a 4 m intercalation of fractured breccia along a fault. The lower Proterozoic Kesuer Group limestone forms the La Bei fault zone, with a lithology dominated by fractured breccia and locally complete thin layers of limestone. The exposed thickness is 1177.0 m. Continuous temperature measurements in the YST1 geothermal well indicate an average geothermal gradient of 1.69 °C/100 m. Geothermal anomalies are mainly distributed within the upper 320 m of the well, where the temperature gradient reaches 7.26 °C/100 m. From 320 to 1380 m, the average temperature gradient is only 0.165 °C/100 m. It is evident that the main heat sources in the YST1 geothermal well are concentrated in the fault-fractured zone in the upper part of the well. This is due to the fact that the YST1 geothermal well passes through the La Bei Fracture Zone, resulting in a rapid warming of the geothermal water and reaching a steady rise in water temperature at about 300 m, after which there is no significant change until the bottom of the well, and the geothermal gradient is very small.

### Hydrogeochemical simulation

Based on the analysis of hydrogen and oxygen stable isotopes, geothermal water in the study area is primarily recharged by atmospheric precipitation, with evident water–rock interactions occurring during the infiltration process. Precipitation infiltrates in the mountainous region of southern Laji Mountain and becomes heated near deep faults through fractures in the rock strata. It then migrates northward along NNE-trending extensional faults, sequentially passing through the fractured zones of Mesoproterozoic Kesuer Formation limestones and schists, as well as the calcareous slate and phyllite of the Qingshipo Formation, before reaching the deep geothermal reservoir. Accordingly, two simulation flow paths were constructed for the hydrogeochemical modeling. Both originate from rainwater sample HZ1, and terminate at geothermal well water samples YST1 and YST2, respectively. These simulations aim to reveal the hydrogeochemical reactions occurring along the subsurface circulation pathway after meteoric recharge. The selected mineral phases involved in the modeling include gypsum, kaolinite, halite, calcite, dolomite, Ca-montmorillonite, quartz, NaX, and CaX_2_.

The simulation results are summarized in Table 2. Along both flow paths (HZ1 → YST1 and HZ1 → YST2), Ca-montmorillonite was precipitated with significant mass transfer, reaching − 87.070 × 10^–3^ mmol/L along HZ1 → YST1. In contrast, gypsum, kaolinite, halite, calcite, dolomite, and quartz underwent dissolution, with respective dissolution quantities to groundwater of 0.733 × 10^–3^, 101.400 × 10^–3^, 0.126 × 10^–3^, 17.500 × 10^–3^, 3.853 × 10^–3^, and 116.800 × 10^–3^ mmol/L. Among these, kaolinite and quartz exhibited the highest dissolution magnitudes. Cation exchange reactions between groundwater and surrounding rocks occurred in the forward direction, resulting in an increase in Na^+^ concentration (0.560 × 10^–3^ mmol/L) and a corresponding decrease in Ca^2+^ (0.280 × 10^–3^ mmol/L).

**Table 2:** Reverse hydrogeochemical simulation results.

Mineralogical phase	Transfer volume (10–3 mmol/L)	
	HZ1 → YST1	HZ1 → YSR2
Gypsum	0.733	0.296
Kaolinite	101.400	62.360
Halite	0.126	0.079
Calcite	17.500	10.200
Dolomite	3.853	3.418
CaX_2_	− 0.280	− 0.046
NaX	0.560	0.092
Ca-Montmorillonite	− 87.070	− 53.530
Quartz	116.800	71.860

A similar pattern of mineral dissolution and precipitation was observed along the HZ1 → YST2 pathway. Ca-montmorillonite precipitation reached − 53.530 × 10^–3^ mmol/L, while dissolution quantities of gypsum, kaolinite, halite, calcite, dolomite, and quartz were 0.296 × 10^–3^, 62.360 × 10^–3^, 0.079 × 10^–3^, 10.200 × 10^–3^, 3.418 × 10^–3^, and 71.860 × 10^–3^ mmol/L, respectively. Cation exchange processes resulted in a Na^+^ release of 0.092 × 10^–3^ mmol/L and a Ca^2+^ decrease of 0.046 × 10^–3^ mmol/L.

The dissolution of kaolinite and quartz released Al^3+^ and SiO_2_ (aq). However, due to the tendency of Al^3+^ to precipitate or form complexes under most groundwater conditions, extensive precipitation of Ca-montmorillonite was observed along both flow paths. The dissolution of calcite and dolomite contributed substantial amounts of Ca^2+^, Mg^2+^, and HCO_3_^−^ to the geothermal water. The widespread dissolution of carbonate minerals is closely associated with the distribution of dolomitic carbonate rocks of the Kesuer Formation in the study area. These lithologies are highly susceptible to dissolution under geothermal fluid influence, leading to significantly elevated concentrations of Ca^2+^ Mg^2+^, and HCO_3_^−^ in the groundwater, ultimately resulting in a hydrochemical type dominated by HCO_3_^-^ as the major anion and Ca^2+^ and Mg^2+^ as the dominant cations.

### The genesis model of geothermal water

#### Heat source

Based on the results of geophysical exploration and the temperature curve from the YST1 geothermal well, the Quaternary thickness in the Yaoshuitan geothermal field is relatively thin, with a shallow cap rock. The geothermal reservoir is mainly composed of the fractured zones along the La Bei fault. The geothermal gradient is relatively high within 300 m, reaching the wellhead temperature, reflecting the characteristics of the geothermal gradient in fault zone areas. The shallow depth of the crust in the region leads to a shallower depth of the underlying soft flow layer, making it easier for magma materials from the shallow soft flow layer to intrude into the shallow crust along regional deep faults. This causes an increase in the geothermal gradient in the shallow crust, forming an abnormal geothermal zone, which is the primary heat source for the thermal water in the Yaoshuitan geothermal field.

#### Cap rock

**Fig. 9:**
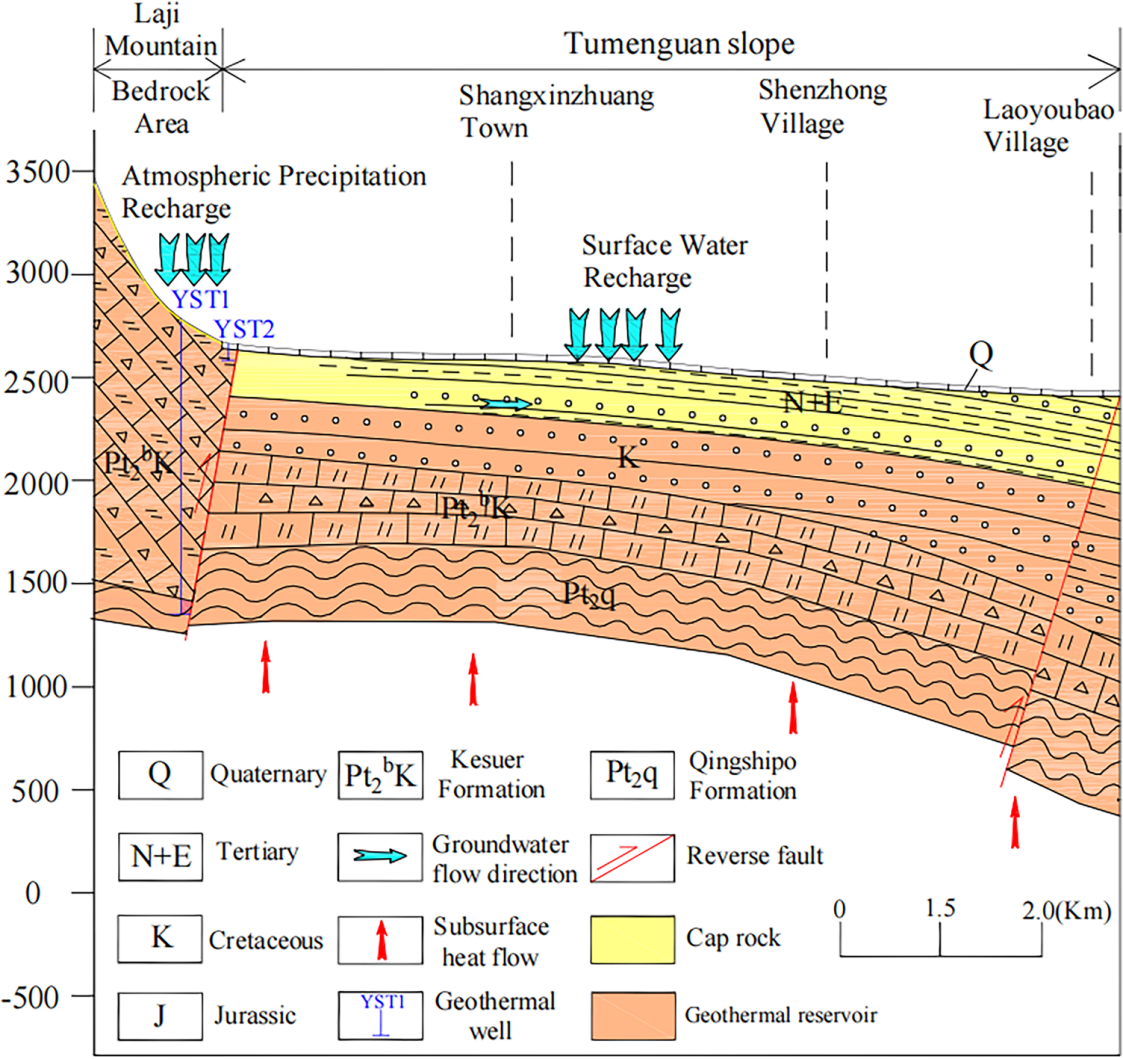
Genesis Model of Geothermal Water in Yaoshuitan, Huangzhong District. The map was drawn using AutoCAD 2018 (https://cad.autodesk-cn.com/).

The cap rock is composed of impermeable or weakly permeable layers that directly cover the geothermal reservoir, serving as an insulator to retain heat. However, the Yaoshuitan geothermal field located in the foreland area to the south of the deep fault on the northern slope of the Laji Mountain, within an uplifted bedrock region, and lacks an effective cap rock to retain heat. According to the core results from the YST1 geothermal well, the exposed formations in the geothermal wellbore are as follows: the upper Quaternary Upper Pleistocene (Q_4_^apl^) (0.0–8.0 m), consisting of a thin yellowish-gray loess layer on top and a black silty gravel layer at the bottom; the Proterozoic Kesuer Formation limestone (Pt_2_bK) (8.0–103.0 m), characterized by grayish-white, blocky structure; the Proterozoic Kesuer Formation limestone (Pt_2_bK) (103.0–1280.0 m), mainly composed of fractured breccia, with some well-preserved thin layers of limestone in local sections; and the Proterozoic Kesuer Formation schist (Pt_2_bK) (1280.0–1403.46 m), with a brown color and foliated structure. However, due to the region’s location in the uplifted mantle zone, and the development of longitudinal joints and fault structures associated with the Xining anticline, the geothermal heat flow is released upward along the pathways formed by deep, large faults. As shown in Fig. 9, both the YST1 and YST2 geothermal wells extend to the fault zone, resulting in wellhead temperatures of 41 °C in both wells.

#### Geothermal reservoir

The primary thermal reservoir in the study area is the carbonate thermal reservoir of the Kesuer Formation in the Jixian Group. This reservoir mainly consists of dolomitic crystalline limestone and dolomite from the Kesuer Formation. The ancient karst features in this stratigraphic unit are highly developed, with fragmented rock masses, well-developed joints and fractures, and dense fault structures. Among these, the light-gray dolomitic crystalline limestone and gray and grayish-white dolomite exhibit karst caves that are flat-oval in shape, with the long axis parallel to the bedding planes. The long axis ranges from 1.50 to 8.00 m, and the short axis from 0.50 to 6.00 m. Core samples show the development of dissolution pores and caves, and pyrite alteration is observed in the fractures and joints. As a result, fissure-cave water is present within the carbonate rocks, with substantial water volume, high water quality, and a mineralization of less than 1 g/L. In the Yaoshuitan geothermal field, the lithology of the thermal reservoir is composed of intact rock (roof), fractured zone, fault clay (lime-like), fractured zone, and intact rock. Additionally, the thermal reservoir type in the Yaoshuitan geothermal field is of the fault zone convective type, which leads to similar water temperatures in two geothermal wells: YST1, with a depth of 1403.46 m, and YST2, with a depth of 52.68 m. This indicates that the temperature increase rate of the fault zone convective type is not as pronounced as that of the basin conductive type.

#### Establishment of geothermal genesis models

Through an analysis of the chemical composition, recharge sources, recharge elevation, reservoir temperature, and circulation depth of the Yaoshuitan geothermal field, the characteristics of geothermal water resources in the study area, which are associated with an uplifted fault-type geothermal system, are as follows: (1) The heat source is derived from the heat flow of the lower crust and upper mantle. The deep, large fault of the Laji Mountain serves as a thermal conduit, with heat rising and diffusing along the fault primarily through convection. The average geothermal gradient is 1.69 °C/100 m, with an indistinct temperature increase in the strata, characteristic of a typical fault zone temperature anomaly. (2) Since the Yaoshuitan geothermal field located in the foreland area south of the deep faults on the northern slope of Laji Mountain, it belongs to an uplifted bedrock mountainous region. The area exhibits developed extensional joints and faults associated with the Xining anticline, and lacks an effective cap rock for thermal insulation. (3) The geothermal reservoir is primarily composed of carbonate rocks from the Kesuer Formation of the Jixian Group, mainly consisting of dolomitic crystalline limestone and dolomite. The reservoir type is classified as fault-zone convection type. (4) The underground geothermal water is primarily replenished by atmospheric precipitation from the southern Laji Mountain region, at elevations between 3619.52 and 3797.52 m. After being heated through fractures in the strata near deep faults, the water flows northward along an NNE-trending extensional fault. The carbonate fault fracture zones, connected karst fractures, and caves serve as both the geothermal water flow and heat storage pathways. Geothermal water flow is blocked by NW-trending compressive shear faults, which lead to its enrichment in fault zones. The circulation depth is 3756.69 m. (5) The geothermal water is identified as confined groundwater within fractured bedrock aquifers associated with fault zones. Along its circulation pathway, groundwater sequentially traverses the fractured belts of Mesoproterozoic Kesuer Formation limestones and schists, followed by the calcareous slate and phyllite of the Qingshipo Formation, before ultimately entering the geothermal reservoir. During subsurface migration, extensive water–rock interactions occur between the groundwater and the surrounding lithologies. The dissolution of dolomite and calcite plays a dominant role in the enrichment of Ca^2+^, Mg^2+^, and HCO_3_^−^. Calcite dissolution primarily contributes Ca^2+^ and HCO_3_^−^, whereas dolomite dissolution further reinforces the dominance of these ions and significantly enhances Mg^2+^ concentrations. As a result, a hydrochemical type characterized by HCO_3_-Ca·Mg type groundwater is developed in the geothermal system. (6) The water temperature is 41°C, categorizing it as a small low-temperature geothermal field. Based on the above, the formation model for the uplifted fault-type geothermal water in the study area is concluded (Fig. 9).

## Conclusion

1. The water temperature of both YST1 and YST2 geothermal wells is 41°C, classifying them as low-temperature geothermal fields. The dominant cations are Ca^2^⁺ and Mg^2^⁺, with HCO_3_^−^ as the predominant anion. The water type is classified as HCO_3_-Ca·Mg, featuring a mineralization (TDS) ranging from 2.178 to 2.338 g/L. The pH values, ranging from 6.94 to 7.82, indicate mildly saline to near-neutral water conditions.
2. The thermal reservoir temperatures of the YST1 and YST2 geothermal wells are 56.52 °C and 67.34 °C, respectively, with circulation depths of 3756.69 m. According to the analysis of hydrogen and oxygen isotopes (δD, δ^18^O), the underground thermal water in the study area is primarily recharged by atmospheric precipitation from the southern Laji Mountain area at elevations ranging from 3619.52 m to 3797.52 m. Additionally, the deep underground thermal water is influenced by secondary faults along the longitudinal direction, leading to a younger age and lower temperatures at greater depths.
3. The study area exhibits an uplifted fault-type geothermal system, where the heat source originates from lower crust and upper mantle heat flow. Deep-seated faults in the Laji Mountain act as primary heat-conduction pathways, facilitating upward migration and lateral diffusion of thermal energy, primarily through convective circulation. The Yaoshuitan geothermal field is situated in the foothill zone south of these northern slope faults, within an uplifted bedrock domain. The shallow structure is characterized by vertical extension joints and fault systems associated with the Xining anticline, lacking an effective thermal insulation cap layer. The thermal reservoir is principally composed of carbonate rocks from the Jixian System Kesuer Formation, including dolomitic crystalline limestone and dolomite. During groundwater circulation, intensive water–rock interactions with these lithologies drive dissolution of calcite and dolomite, resulting in the HCO_3_-Ca·Mg hydrochemical type dominance. Thermal water is heated as it flows through fracture networks near deep faults, then migrates northward along NNE-striking extensional faults. Carbonate fault zones, interconnected karst fissures, and solution caves serve as both flow pathways and heat storage media. Flow is impeded by NW-striking compressional-shear faults, causing thermal water accumulation along these structural barriers. Notably, the fault-zone convection-type reservoir exhibits a muted temperature gradient: the 1403.46 m depth in YST1 shows a temperature similar to 52.68 m in YST2. This contrasts with basin-conductive reservoirs, where temperature gradients are typically more pronounced, highlighting the distinct thermal behavior of fault-dominated circulation systems.

## Data Availability

The data used to support the findings of this study are included within the article. It can be obtained from the first author upon request.

## References

[1] Mongillo, M. A. & Axelsson, G. Preface to geothermics special issue on sustainable geothermal utilization. *Geothermics* **39**(4), 279–282 (2010).

[2] Barbier, E. Nature and technology of geothermal energy: A review. *Renew. Sustain. Energy Rev.* **1**(1), 1–69 (1997).

[3] Han, S. et al. Research on the strengthening of geothermal resource exploration, development, and utilization in Qinghai. *Drill. Eng.* **40**(03), 80 (2013).

[4] Zhao, Z. et al. Preliminary study on the overview and exploration, development, and utilization planning of geothermal resources in Qinghai Province. *J. Qinghai Environ.* **23**(03), 130–135 (2013).

[5] Lang, X. Thermal structure and geothermal genesis mechanism of the guide basin. *Chin. Acad. Geol. Sci.* **38**, 43–46 (2016).

[6] Li, L. Study on the distribution pattern and genesis model of geothermal resources in the guide basin, Qinghai. *East China Univ. Technol.* **76**, 1–19 (2016).

[7] Wang, H. et al. Analysis of geothermal resource characteristics and utilization in the Gonghe region, Qinghai Province. *China Mining Magazine* **32**(06), 167–174 (2023).

[8] Li, Y. Hydrogeochemical characteristics and genesis analysis of groundwater in the Qiaobei-Qia region, Gonghe Basin, Qinghai Province. *East China Univ. Technol.* **13**, 2403 (2016).

[9] Lu, Z. et al. Hydrogeochemical characteristics and genesis of geothermal fluids in the northern Zhaoqiu area. *Carsologica Sinica* **43**(01), 12–24 (2024).

[10] Du, X. et al. Chemical characteristics and genesis model of geothermal water in the Qingshankou formation, Daqingzi well area, Southern Songliao Basin. *Bullet. Geol. Sci. Technol.* **43**(03), 22–35 (2024).

[11] Deng, J. et al. Hydrochemical characteristics and genesis of geothermal water in Shuiyindong, Guizhou. *Acta Mineralogica Sinica* **41**(03), 355–366 (2021).

[12] Avşar, Ö. & Altuntaş, G. Hydrogeochemical evaluation of Umut geothermal field (SW Turkey). *Environ. Earth Sci.* **76**(16), 582 (2017).

[13] Landa-Arreguín, J. F. A. et al. Hydrogeochemistry and isotopic assessment of a new geothermal prospect in Rancho Nuevo (Guanajuato-Central Mexico)[J]. *J. Geochem. Explor.* **253**, 107294 (2023).

[14] Gao, N. et al. Genesis model of the geothermal system in the Shulu Sag, Jizhong depression. *Geol. J. China Univ.* **28**(06), 920–932 (2022).

[15] Yan, B. et al. Characteristics and genesis model of basin-type geothermal water in the basaltic area of Changbai Mountain. *Geol. Rev.* **64**(05), 1201–1216 (2018).

[16] Mo, Y. et al. Survey and prospect analysis of geothermal resources in the northern region of Liuzhou, Guangxi. *Mineral Resour. Geol.* **35**(06), 1102–1110 (2021).

[17] Wang, X., Zhang, L. & Li, S. Genesis model analysis of the Yangmei Chong geothermal field, Guangxi. *Carsologica Sinica* **43**(04), 876–888 (2024).

[18] Song, M. et al. Hydrogeochemical characteristics and genesis of the Chazi geothermal field area in Tibet. *Geofluids* **20**, 5815996 (2022).

[19] Zhu, H. Study of basin-type geothermal resources in the northern Songliao basin. *Northeast Petrol. Univ.* **225**, 120362 (2011).

[20] Li, H. et al. Preliminary study on the hydrochemical characteristics and thermal water origin of the Yaoshuizha geothermal field Xining. *Acta Geol. Sin.* **09**, 1299–1304 (2007).

[21] Craig, H. Isotopic variations in meteoric waters. *Science* **133**(3465), 1702–1703 (1961). 17814749 10.1126/science.133.3465.1702

[22] Tan, H., Zhang, W., Chen, J., Jiang, S. & Kong, N. Isotope and geochemical study for geothermal assessment of the Xining basin of the Northeastern Tibetan Plateau. *Geothermics* **42**, 47–55 (2012).

[23] Wang, Y. et al. Estimation of subsurface heat storage temperature using geothermal temperature scale. *Geoscience* **04**, 605–612 (2007).

